# Efficacy of Voriconazole Corneal Intrastromal Injection for the Treatment of Fungal Keratitis

**DOI:** 10.1155/2021/5597003

**Published:** 2021-07-30

**Authors:** Chenshuang Li, Kunpeng Pang, Liqun Du, Xinyi Wu

**Affiliations:** Qilu Hospital, Cheeloo College of Medicine, Shandong University, Jinan, Shandong, China

## Abstract

**Purpose:**

To evaluate efficacy and safety of novel tricyclic corneal stroma injection (TCSI) voriconazole for the treatment of fungal keratitis.

**Methods:**

This retrospective cohort study included data of 57 patients (57 eyes) with fungal keratitis. The TCSI group consisted of 27 patients (27 eyes) who were injected voriconazole once via TCSI procedure within one week after enrollment, in addition to conventional antifungal treatment. The control group consisted of 30 patients (30 eyes) who were treated using conventional antifungal treatment modalities. The outcome measures consist of the 3-week and 3-month best-corrected visual acuity (BCVA) values and size of infiltrate or scar, time to re-epithelialization, corneal perforation rate and/or therapeutic penetrating keratoplasty (TPK) requirement, the preoperative and post-TCSI corneal endothelial cell density (ECD), and the intraocular pressure (IOP) of the treated eye and the respective contralateral eye.

**Results:**

There were no significant differences in the baseline demographic and clinical characteristics between the two groups. 3 weeks and 3 months after enrollment, the TCSI group exhibited an increase in visual acuity (*P* < 0.05), and there was no significant difference in the size of infiltrate or scar between two groups (*P* > 0.05). Time to re-epithelialization was shorter in the TCSI group than in the control group (*P* < 0.05). There was no statistically significant difference between corneal ECD on the day before and 7 days after TCSI and the IOP of treated and contralateral healthy eyes on the day before and 1 day, 3 days, 7 days, and 1 month after TCSI (*P* > 0.05). The difference in the risk of perforation and/or TPK requirement was not statistically significant between two groups (*P* > 0.05).

**Conclusion:**

Localized injection of voriconazole using TCSI may be a minimally invasive, safe, and effective adjuvant treatment modality for fungal keratitis.

## 1. Background

Fungal keratitis is one of the most serious infectious diseases of the eye that usually leads to blindness. Trauma, indiscriminate use of corticosteroids and antibiotics, and prolonged contact lens use have resulted in a tremendous increase in the ocular morbidity of fungal keratitis, especially in developing countries [1–4]. Two classes of antifungal agents are currently used for treating fungal keratitis: polyenes (natamycin, amphotericin B) and azoles (voriconazole) [5–7]. However, the antifungal effect of these drugs in the treatment of fungal keratitis is compromised owing to the poor penetration into the corneal stroma, which is usually responsible for delayed ulcer healing, recurrence of fungal infection, corneal scar formation, chronic local inflammation, and progressive thinning of the corneal stroma or even perforation [8–10]. Therefore, most patients with fungal keratitis eventually need corneal transplantation. Paradoxically, the scarcity of corneal donors, high cost of corneal transplantation surgery, and poor or nonadherence to immunosuppressive medication post-transplantation limit its application in developing countries [11]. Hence, it is important to find a new effective strategy to combat fungal infections, alleviate stromal inflammation and scar formation, and ultimately avoid emergency keratoplasty. Voriconazole, a second-generation triazole agent, has been used to treat aspergillosis and *S. apiospermum* and *Fusarium* spp. infections [12]. It exhibited a higher permeability in the corneal stroma than other antifungal agents, but its ability to reach the corneal tissue is still limited secondary to ocular drug delivery challenges [13]. Especially, it cannot effectively destroy the fungi growing in the deep corneal stroma, which ultimately results in prolonged treatment and high recurrence [12–15]. Several researchers implemented the corneal stroma injection procedure to increase the local drug concentration in the corneal lesion and observed its efficacy to solve the issue of poor penetrability of antifungal agents into the corneal stroma. Although studies have reported that intrastromal voriconazole injection could increase the drug concentration in the cornea stroma, they lacked consensus on its efficacy for treating fungal keratitis [16–20]. These differences may be attributed to the lack of uniformity in the injection method used in various studies, which resulted in variable drug concentration and distribution in the corneal stroma. At the same time, some studies have suggested that intrastromal injections were dangerous and increased the risk of corneal perforation [21]. Thus, the tricyclic corneal stroma injection (TCSI) procedure was designed for intrastromal injection of voriconazole to overcome this dilemma. This study aimed to evaluate the efficacy and safety of localized injection of voriconazole with the TCSI procedure for the treatment of fungal keratitis.

## 2. Methods

### 2.1. Participants and Design

This retrospective cohort study included data from 57 patients (57 eyes) with fungal keratitis who were enrolled at the Qilu Hospital of Shandong University between February 2015 and December 2019. The TCSI procedure was designed in June 2018. Between February 2015 and June 2018, all patients who met the inclusion criteria were treated with conventional antifungal treatment only, as the control group. Between June 2018 and December 2019, all patients who met the inclusion criteria were injected voriconazole once via TCSI procedure within one week after enrollment, in addition to receiving treatment with conventional antifungal treatment, and these patients were classified as the TCSI group. The TCSI group consisted of 27 patients (27 eyes), while the control group consisted of 30 patients (30 eyes).

The inclusion criteria for this study were as follows: (1) patients who tested positive for fungal infections on corneal smear examination or in vivo confocal microscopy (IVCM) [4]; (2) corneal infiltration area measuring less than 10 mm in diameter; and (3) follow-up for 3 months or more after discharge. The exclusion criteria for this study were as follows: (1) mixed keratitis; (2) corneal perforation or impending perforation; (3) corneal lesions involving the sclera; (4) concomitant endophthalmitis; (5) presence of other ocular diseases, such as glaucoma and iridocyclitis; and (6) history of corneal transplantation.

### 2.2. Interventions and Outcome Measures

**Figure 1:**
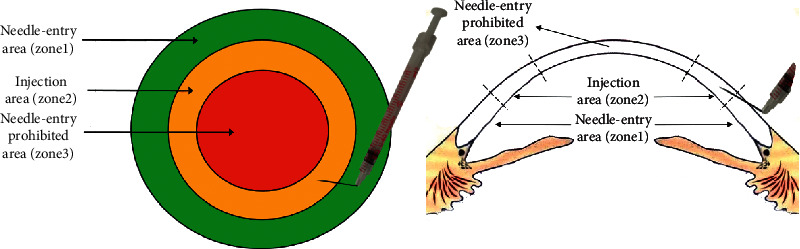
The diagram of TCSI procedure.

The TCSI procedure was designed such that the cornea was divided into three circular areas as shown in Figure 1: the needle-entry area (zone 1), injection area (zone 2), and “needle-entry prohibited” area (zone 3).

The annular area adjacent to the corneal limbus measuring 2 mm in width was designated as zone 1. The area with a diameter of 5–6 mm at the center of the cornea was designated as zone 3, while the area between zones 1 and 3 was designated as zone 2. A 0.5 mg/mL solution of voriconazole (provided by Jincheng Haisi Pharmaceutical Co., Ltd., reconstituted with lactated Ringer's solution) was prepared before the procedure. The needle of a 29-G syringe was bent at 90° perpendicular to the barrel, with the needle bevel upward. The bent needle was inserted slowly to 50% of the depth of the corneal stroma at an angle of 15° to the corneal surface in zone 1 and cautiously inserted parallel to the cornea lamella in zone 2 followed by slow injection of the drug solution into the corneal stroma, until the corneal stroma around the lesion appeared to be swollen and cloudy. The procedure should be performed in the 4–6 different regions with 0.1–0.15 mL administered at each point to ensure that the entire corneal stroma around the lesion was swollen and cloudy. For eccentric lesions, the needle-entry area remained in zone 1. The needle-entry point selected was close to the lesion, and the surgeon appropriately increased the injection pressure to make sure that the entire corneal stroma around the lesion was swollen and cloudy. TCSI was performed after surface infiltration anesthesia in the operating room by the same operator (who was not involved in data and statistical analysis).

The control group was treated by conventional methods including topical voriconazole 10 mg/mL (every 2 h) (provided by Jincheng Haisi Pharmaceutical Co., Ltd., reconstituted with lactated Ringer's solution) and topical natamycin 50 mg/mL (every 2 h) (provided by North China Pharmaceutical Co., Ltd).

The TCSI group was treated by conventional methods in addition to TCSI with voriconazole once within one week after enrollment.

The healing of keratitis was considered the complete re-epithelialization with the complete resolution of the corneal infiltrates and no hyphae growth in IVCM examination at the same time. And topical antifungal therapy was continued 4 times a day for 2 weeks after the healing.

The baseline demographic characteristics, clinical characteristics, microbial culture results, and treatment outcomes of the control and TCSI groups were evaluated and compared. The demographic characteristics included age, sex, occupation, affected eye, presence/absence of systemic disease, and use of medication before enrollment. The initial clinical characteristics included symptom duration, lesion location, size of the infiltrate or scar and the epithelial defect, depth of infiltration, presence of hypopyon, and preoperative best-corrected visual acuity (BCVA) [expressed in logarithm of the minimum angle of resolution (LogMAR)]. The duration of symptoms was defined as the interval between the onset of symptoms and the initial visit. The corneal ulcers were classified into central or peripheral lesions based on the half radius of the cornea [22]. The size of the infiltrate/scar and epithelial defect size were calculated by geometric mean of longest diameter and longest perpendicular, respectively [23]. The depth of infiltration divided into three categories: 0% to 33%, 33% to 67%, and 67% to 100% [24]. Visual acuity was recorded using the international standard Snellen visual acuity chart and converted into LogMAR for statistical analysis. Light perception, hand movement, and counting fingers were designated as 2.6, 2.4, and 2.0 LogMAR, respectively [25]. The treatment outcomes were evaluated using the 3-week and 3-month BCVA values and size of infiltrate or scar, time to re-epithelialization, rate of corneal perforation, and/or therapeutic penetrating keratoplasty (TPK) [20, 24]. Re-epithelialization was defined as the absence of an epithelial defect on fluorescein staining (fluorescein sodium stain strips were provided by Liaoning Meizilin Pharmaceutical Co., Ltd) [26]. To evaluate the safety of TCSI procedure, the preoperative and post-TCSI corneal endothelial cell density (ECD) were evaluated in patients with a relatively mild edema. The ECD was measured at the same position for each case using IVCM (HRT-3, Heidelberg, Germany). Moreover, the preoperative intraocular pressure (IOP) and 1-day, 3-day, 7-day, and 1-month post-TCSI IOP of the treated eye were compared with that of the respective contralateral eye. IOP was measured using a noncontact tonometer (NF-510; Nidek, Japan) in patients with a relatively clear cornea.

### 2.3. Statistical Analysis

The baseline characteristics of the two groups were compared using Fisher's exact test for categorical variables and Wilcoxon's rank-sum test for continuous variables. Multiple linear regression was used to analyze BCVA and size of the infiltrate or scar measured at 3 weeks and 3 months after treatment with pretreatment measurements as covariates. The time to re-epithelialization was analyzed using multiple linear regression with epithelial defect size as the covariate. Paired *t*-tests were used to compare the ECD recorded on the day before and 7 days after TCSI. Independent *t*-tests were used for comparing the IOP of the treated and contralateral healthy eye before and 1 day, 3 days, 7 days, and 1 month after TCSI. A Cox proportional hazards regression model was generated with covariates for the group and baseline infiltrate depth to assess the odds of corneal perforation and/or TPK. *P* < 0.05 (two-tailed) was considered statistically significant.

The last BCVA observed was carried forward or was considered to be 1.7 LogMAR if there were no “last observation” data available, in case the BCVA measured after TPK was missing. The last observation (before TPK) was carried forward, in case of missing data on the infiltrate or scar resulting from TPK [24].

All analyses were conducted using SPSS 19.0 statistical software.

### 2.4. Ethical Approval

The Ethics Committee of Qilu Hospital of Shandong University granted ethical to this study, which was performed according to the principles of the Declaration of Helsinki (NO : KYLL-2020-620). All study participants provided written informed consent.

## 3. Results

Fifty-seven patients with fungal keratitis were included in this study. 30 patients (30 eyes) were placed in the control group, and 27 patients (27 eyes) were placed in the TCSI group. All participants were enrolled at the Qilu Hospital of Shandong University. No major differences were identified between the baseline demographics, clinical characteristics, and microbial culture results of the two groups, which are outlined in Table 1.

Although the patients have tested positive for fungal infections on corneal smear examination or IVCM, the microbiological culture results were different. Organisms isolated from baseline cultures are described in Table 2. Eighteen culture samples (32%) tested positive for the *Fusarium* species, 5 (9%) for *Alternaria* Nees, 3 (5%) for the *Aspergillus* species, and 3 samples (5%) tested positive for other fungi. The culture results were negative in 28 samples (49%). In total, 28 negative samples, 20 were confirmed by both cornea smear examination and IVCM, 7 were confirmed only by IVCM, and 1 was confirmed only by corneal smear examination. There was no significant difference in the kind of fungal strain that patients infected between the two groups (*P*=0.24).

We compared the BCVA of the control (30 eyes) and TCSI (27 eyes) groups. The mean 3-week visual acuity was 0.91 ± 0.87 LogMAR in the TCSI group and 1.33 ± 0.85 LogMAR in the control group. The mean 3-month visual acuity was 0.81 ± 0.84 LogMAR in the TCSI group and 1.28 ± 0.91LogMAR in the control group. Multiple linear regression was used to analyze BCVA. Compared with the control group, the TCSI group had 0.13 LogMAR better visual acuity at 3 weeks after controlling for baseline visual acuity (95% CI, −0.40 to −0.05 LogMAR; *P*=0.01) and had 0.16 LogMAR better visual acuity at 3 months after controlling for baseline visual acuity (95% CI, −0.49 to −0.07 LogMAR; *P*=0.01).

We compared the infiltrate or scar size of the control (30 eyes) and TCSI (27 eyes) groups. Multiple linear regression models also found a 0.12 mm and 0.07 mm reduction in the infiltrate or scar size after 3 weeks (95% CI, −1.11 to 0.11 mm; *P*=0.10) and 3 months (95% CI, −0.86 to 0.35 mm; *P* = 0.40), respectively, in the TCSI group compared with the control group after controlling for baseline values. This difference was not statistically significant.

We compared the time to re-epithelialization of the control (30 eyes) and TCSI (27 eyes) groups. The mean time to re-epithelialization was 17.67 ± 10.27 days in the TCSI group and 25.10 ± 11.42 days in the control group. The TCSI group exhibited a 0.27-day reduction in the time to re-epithelialization after controlling for baseline epithelial defect size (95% CI, −10.75 to −1.30 days; *P*=0.01).

The ECD and IOP were examined during treatment to evaluate the biosafety of the TCSI procedure. In eight patients with a relatively mild edema in the TCSI group, ECD values were measured using IVCM and the preoperative and postoperative ECD at the same position for each case were compared. There was no significant difference between the preoperative and postoperative ECD [2231.88 ± 649.02 mm^2^ and 2189.50 ± 639.20 mm^2^ (*P*=0.90), respectively]. Moreover, the IOP of 5 cases with a relatively clear cornea was measured using a noncontact tonometer. The mean IOP before, 1 day, 3 days, 7 days, and 1 month after the TCSI procedure were 15.18 ± 2.72, 14.2 ± 3.06, 14.34 ± 3.11, 14.04 ± 3.14, and 15.60 ± 2.89 mmHg in the treated eye and 14.82 ± 2.76 (*P* = 0.84), 15.18 ± 2.60 (*P* = 0.60), 13.80 ± 3.01 (*P* = 0.79), 13.94 ± 2.70 (*P* = 0.96), and 15.50 ± 2.62 (*P* = 0.96) mmHg in the contralateral healthy eye, respectively. There was no statistically significant difference between the preoperative and postoperative IOP (1 day, 3 days, 7 days, and 1 month after TCSI) (*P* ≥ 0.05) of the treated and contralateral eyes.

In the TCSI group, six patients received two or more intrastromal injections, including one patient who was injected two times, four patients three times, and one patient four times. All patients' corneal edema disappeared within 24 h in each case. The healthy corneal tissue around the lesion area appeared transparent after repeated injections and showed no abnormalities compared with patients treated with a single injection.

Two patients (4%) from the control group experienced full-thickness corneal perforation. Three patients (5%) from the control group eventually required TPK including the two patients who experienced full-thickness corneal perforation. There was no adverse event in the TCSI group. The Cox proportional hazards model revealed that patients in the TCSI group were less likely to have perforation or transplantation after controlling for baseline infiltrate depth, but this difference was not statistically significant (odds ratio = 0.002; 95% CI, 0 to 33.03; *P* = 0.21).

**Figure 2:**
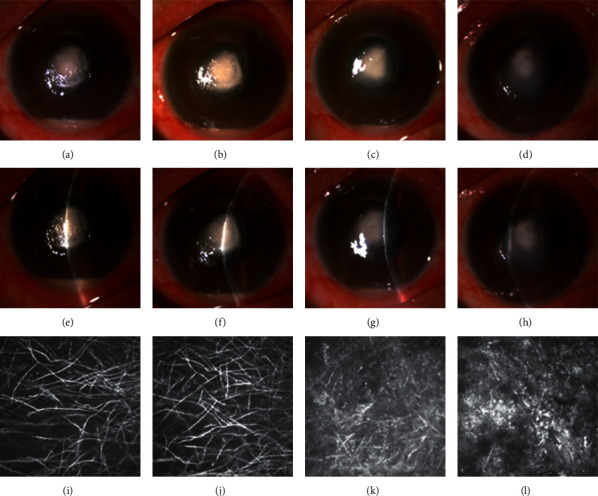
Case 1. (a, e) Anterior segment photograph at enrollment. (b, f) Anterior segment photograph before TCSI (after 5-day conventional antifungal treatment). (c, g) Anterior segment photograph 3 days after TCSI. (d, h) Anterior segment photograph when the patient has been healed. (i) IVCM examination at enrollment. (j) IVCM examination before TCSI (after 5-day conventional antifungal treatment). (k) IVCM examination 3 days after TCSI. (l) IVCM examination when the patient has been healed.

**Figure 3:**
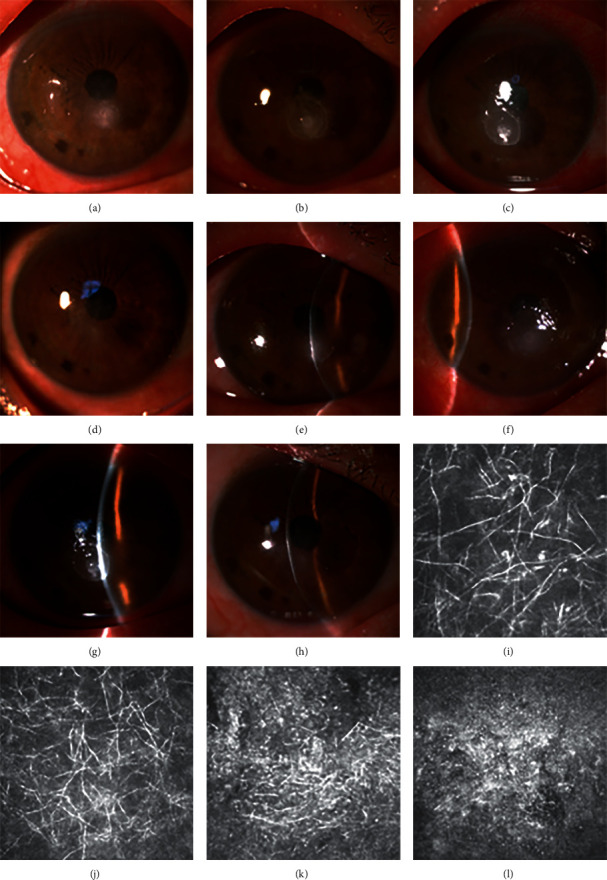
Case 2. (a, e) Anterior segment photograph at enrollment. (b, f) Anterior segment photograph before TCSI (after 5-day conventional antifungal treatment). (c, g) Anterior segment photograph 3 days after TCSI. (d, h) Anterior segment photograph when the patient has been healed. (i) IVCM examination at enrollment. (j) IVCM examination before TCSI (after 5-day conventional antifungal treatment). (k) IVCM examination 3 days after TCSI. (l) IVCM examination when the patient has been healed.

We found swelling, broken, and dissolved hyphae in the corneal stroma of the TCSI group using IVCM examination 3 days after TCSI (Figures 2 and 3), which nearly had no change after 5 days of conventional antifungal treatment. At the same time, we observed significant improvement of clinical signs 3 days after TCSI.

## 4. Discussion

Voriconazole is a triazole antifungal agent that induces fungal death by inhibiting lanosterol 14*α*-demethylase in the fungal cell membrane, which interrupts the conversion of lanosterol to ergosterol, thereby affecting the stability of the fungal cell membrane. It is used to treat several fungal infections caused by *Aspergillus*, *Candida*, and *Fusarium* and fungi resistant to fluconazole, itraconazole, or amphotericin B. Voriconazole is a kind of concentration-dependent drug, which means that the higher the concentration reaching the lesion, the stronger the antifungal effect [12, 14, 15]. At the same time, the concentration cannot cause adverse effects on the cornea. Although voriconazole exhibits higher permeability in the cornea stroma than other antifungal agents, but its ability to reach corneal tissue is still limited secondary to ocular drug delivery challenges. This ultimately results in prolonged treatment and high recurrence [13]. Chronic inflammatory cells and cytokine infiltration induced by fungal infection usually lead to scar formation in the stroma, progressive corneal thinning and perforation, or even fungal endophthalmitis. Several researchers implemented the corneal stroma injection procedure to increase the local drug concentration in the corneal lesion and observed its efficacy to solve the issue of poor penetrability of antifungal agents into the corneal stroma. Although studies have reported that intrastromal voriconazole injection could increase the drug concentration in the cornea stroma, the therapeutic efficacy was controversial [16–20]. These controversies may be attributed to the lack of uniformity in the injection method used in various studies, which resulted in variable drug concentration and distribution in the corneal stroma. At the same time, some studies suggested that intrastromal injections may increase the risk of corneal perforation in fungal keratitis [21]. Thus, the tricyclic corneal stroma injection (TCSI) procedure was designed for intrastromal injection of voriconazole to overcome this dilemma.

In other studies, intrastromal injection was defined as administering around the infiltrate to surround the entire circumference of the lesion [16–20]. In the TCSI procedure, we chose zone 1 as the needle-entry area, which was the peripheral cornea adjacent to the limbus, an annular-shaped region with a width of 2 mm and depth of 0.7–1 mm. This region is the thickest part of the cornea that might not easily be penetrated (such as the central cornea). Moreover, needle insertion in this area has little effect on visual acuity. The diameter and thickness of the center of the cornea are 5–6 mm and 0.50–0.59 mm, respectively. It was designated as the “needle-entry prohibited area” because it is the corneal optical center, which is most vulnerable to fungal infection and thus progressive stromal thinning. Needle insertion in this region is accompanied by a high risk of perforation, drug leakage into the anterior chamber, or even the trauma to the lens since the diameter of the 29-G needle (0.33 mm) is similar to the thickness of the thinned stroma. Moreover, the act of piercing the central cornea could damage the stromal lamellae, induce scar formation, and lead to further deterioration in vision. At the same time, the TCSI procedure could ensure complete diffusion of the drug from the corneal periphery to the lesion and significantly improve the efficacy of stromal injection. In China, the majority of fungal keratitis cases are caused by *Fusarium* [3]. *Fusarium* grows and extends parallel to the corneal lamellae within the corneal stroma. The drugs only injected around the lesion cannot spread to all the hyphae growing in the stroma equally and are unable to destroy the fungi in the deep stroma [27]. During the TCSI procedure, the bevel of the needle measuring 1.68 mm in length should be facing upward and inserted into the stroma at a depth that is approximately half of the corneal thickness. The needle should be controlled with an angle of 15° to the corneal surface before the first insertion to avoid the risk of perforation during this process. For eccentric lesions, the needle-entry area remained in zone 1. A needle-entry point was selected, which was close to the lesion, and the surgeon appropriately increased the injection pressure to make sure that the entire corneal stroma around the lesion was swollen and cloudy.

The injection concentration of voriconazole was 0.5 mg/mL in our study. Previous studies reported that an intrastromal injection of voriconazole 0.5 mg/mL would not result in toxic complications in the cornea [28, 29]. We found that stromal edema was transient and disappeared within 24 hours of the injection. And our study confirmed that there was no change in ECD during the follow-up period. According to the literature, this injection concentration achieved the minimum inhibitory concentration for 90% (MIC90) of the most fungi including *Aspergillus* species and *Candida* species, along with many other organisms [30, 31].

Fungal invasiveness is facilitated by the ability of the fungus to produce enzymes that degrade physical barriers and antimicrobial proteins [32]. Mycotoxins can be produced to promote fungal survival in the host [33]. Lectins can inhibit host cell growth and weaken the physical integrity of epithelial cells [34]. Consequently, early elimination of the hyphae present in the corneal tissue is critical to the patient's prognosis. In our study, patients in the TCSI group were injected voriconazole once via TCSI procedure within one week after enrollment. We increased the antifungal drug concentration of the corneal stroma during the early stage of fungal keratitis to kill the fungus quickly, manage the conditions of patients with fungal keratitis, reduce inflammation, promote corneal epithelial healing, rearrange corneal stroma collagen, reduce scar formation, and improve the prognosis of BCVA. We found swelling, broken, and dissolved hyphae in the corneal stroma of the TCSI group using IVCM examination 3 days after TCSI. These features were unchanged after 5 days of conventional antifungal treatment. We simultaneously observed a significant improvement in clinical signs 3 days after TCSI. These findings may support the efficacy of TCSI for the medical treatment of fungal keratitis.

Our study showed that the TCSI procedure could improve the 3-week and 3-month BCVA and shorten the time for re-epithelialization while treating fungal keratitis by multiple linear regression analysis. There was no significant difference between the size of the infiltrate or scar between the two groups. This further showed that TCSI of voriconazole would not increase the infiltration/scar area.

In our study, we found that the patients in the TCSI group were less likely to have perforation or transplantation after controlling for baseline infiltrate depth, but this difference was not statistically significant. Furthermore, the three patients requiring TPK were from the control group, and there was no adverse event in the TCSI group. Although the result was not statistically significant, it indicated that TCSI was a safe procedure. If we enlarge the size of sample, it may lead to a more meaningful result.

Studies have reported that voriconazole entered aqueous humor circulation after intrastromal injection [35]. We were worried that it would produce adverse reactions in the aqueous humor circulation, resulting in abnormal IOP of patients after TCSI of voriconazole. The preoperative IOP and 1-day, 3-day, 7-day, and 1-month post-TCSI IOP of the treated eye were compared with those of the respective contralateral eye. This difference was not statistically significant, which proved that this method was safe and would not impact the IOP.

The pharmacokinetics of voriconazole injected into the corneal stroma is unknown. One study showed that voriconazole injected into the corneal stroma would not remain there for a long time [35]. Thus, repeated injections are required for treating fungal keratitis. Tu suggested that repeated injections of voriconazole into the human corneal stroma were well tolerated, without long-term ocular toxicity [36]. In our study, 6 eyes received two or more intrastromal injections and showed no abnormalities compared with patients treated with a single injection. However, the pharmacokinetics of voriconazole injected into the stroma should be studied further to determine the requisite frequency of corneal stroma injections.

The limitations of this study are as follows: It was a single-center study with a small sample size, and the study was not prospective. More clinical data are required to validate and analyze the efficacy and safety of TCSI. We did not include patients with >10 mm diameter corneal infiltrates and corneal lesions involving the sclera. If the size of the infiltrate was >10 mm, we had to inject the antifungal drug near the lesion. We believe that injecting antifungal drugs near the lesion increases both the corneal pressure and the risk of corneal perforation. Corneal lesions involving the sclera indicate that the sclera has been infected by fungus. And injection of antifungal drugs into the cornea will not kill the fungus in the sclera. At the same time, we have not performed statistical analysis on fungal keratitis derived from different strains.

However, from this retrospective data, localized injection of voriconazole using TCSI could improve the visual acuity or accelerate the epithelial healing of fungal keratitis and exhibited the potentiality to be used for treatment of fungal keratitis. In addition, we did not see any significant difference in change of ECD, IOP, and risk of corneal perforation after stromal injection comparing with the control group. Hence, TCSI might be a minimally invasive and safe adjuvant therapy strategy for fungal keratitis. Multicenter, randomized, prospective trial will be carried out to further validate the efficacy and safety of this procedure in future.

## Figures and Tables

**Table 1:** Baseline demographic and clinical characteristics.

Characteristics	Study group			
	TCSI group (*n* = 27)	Control group (*n* = 30)	Total (*n* = 57)	*P* value^*a*^
*Gender, no.*				
Male	20	16	36	0.17
Female	7	14	21	

*Age, median (IQR), y*	55 (49–65)	54 (45–62)	54 (47–65)	0.63

*Occupation, no.*				
Agriculture	22	24	46	1.00
Nonagriculture^*b*^	5	6	11	
Medication use at enrollment, *no.*^*c*^	16	19	35	0.79

*Etiology, no.*				
Plant trauma	14	11	25	0.16
Others^*d*^	8	6	14	
None	5	13	18	

*Affected eye, no.*				
Right	12	14	26	1.00
Left	15	16	31	

*Visual acuity, median (IQR), LogMAR*	0.90 (0.5–2)	1.1 (0.6–2.4)	1.0 (0.60–2.00)	0.37
*Infiltrate or scar size, median (IQR), mm*	3.9 (2.7–4.7)	3.8 (3.3–5.6)	3.8 (2.9–5.1)	0.44

*Ulcer location, no.*				
Central	24	25	49	0.71
Peripheral	3	5	8	

*Hypopyon, no.*				
None	22	25	47	0.88
<0.5 mm	2	1	3	
≥0.5 mm	3	4	7	

*% of depth, no.*				
>0–33	16	20	36	0.91
>33–67	9	8	17	
>67–100	2	2	4	

*Epithelial defect, median (IQR), mm*	2.5 (1.6–3.5)	2.6 (2.0–3.6)	2.5 (1.8–3.5)	0.55
*Duration of symptoms, median (IQR), d*	20 (7–45)	17.5 (10–30)	20 (10–30)	.80
*Systemic disease, No.* ^*e*^	8	6	14	.54

IQR, interquartile range. ^*a*^The count data were analyzed with Fisher's exact test; the continuous data were analyzed with Wilcoxon's rank-sum test. ^*b*^Includes unemployed, retired, etc. ^*c*^Includes topical ocular antifungals, systemic antifungals, topical antibiotics, lubricating eyedrops. ^*d*^Includes dust, finger, fingernail, sand, insect, iron rod. ^*e*^Includes diabetes mellitus, hypertension, hyperthyroidism, asthma, pulmonary heart disease.

**Table 2:** Microbiological culture results^*a*^.

Organism	TCSI group (*n* = 27)	Control group (*n* = 30)	Total (*n* = 57)
*Fusarium* species	8	10	18
*Aspergillus* species	0	3	3
*Aspergillus flavus*	0	1	1
*Aspergillus fumigatus*	0	1	1
*Aspergillus niger*	0	1	1
*Alternaria* Nees	1	4	5
*Penicillium*	1	1	2
Yeast	1	0	1
Fungal culture negative	16	12	28

^*a*^Comparing species, *P*=0.24 by Fisher's exact test.

## Data Availability

The data sets used and/or analyzed during the current study are available from the corresponding author on reasonable request.

## References

[1] Shah A., Sachdev A., Coggon D., Hossain P. (2011). Geographic variations in microbial keratitis: an analysis of the peer-reviewed literature. *British Journal of Ophthalmology*.

[2] Kredics L., Narendran V., Shobana C. S., Vágvölgyi C., Manikandan P., Group I. H. F. K. W. (2015). Filamentous fungal infections of the cornea: a global overview of epidemiology and drug sensitivity. *Mycoses*.

[3] Sun X.-G., Zhang Y., Li R (2004). Etiological analysis on ocular fungal infection in the period of 1989-2000. *Chinese Medical Journal*.

[4] Thomas P., Kaliamurthy J. (2013). Mycotic keratitis: epidemiology, diagnosis and management. *Clinical Microbiology and Infection*.

[5] Liu L., Wu H., Riduan S. N., Ying J. Y., Zhang Y. (2013). Short imidazolium chains effectively clear fungal biofilm in keratitis treatment. *Biomaterials*.

[6] Sonego-Krone S., Sanchez-Di Martino D., Ayala-Lugo R (2006). Clinical results of topical fluconazole for the treatment of filamentous fungal keratitis. *Graefe’s Archive for Clinical and Experimental Ophthalmology*.

[7] Arora R., Gupta D., Goyal J., Kaur R. (2011). Voriconazole versus natamycin as primary treatment in fungal corneal ulcers. *Clinical & Experimental Ophthalmology*.

[8] Manzouri B., Wyse R. K., Vafidis G. C. (2001). Pharmacotherapy of fungal eye infections. *Expert Opinion on Pharmacotherapy*.

[9] Liu F., Li W., Liu Z., Chen W. (2016). Recent studies on corneal epithelial barrier function. *Chinese Journal of Ophthalmology*.

[10] Awwad S., Mohamed Ahmed A. H., Sharma G (2017). Principles of pharmacology in the eye. *British Journal of Pharmacology*.

[11] Schaub F., Simons H. G., Enders P (2016). Corneal donation: dilemma between growing demand and declining donor rate. *Ophthalmologe*.

[12] Scott L. J., Simpson D. (2007). Voriconazole. *Drugs*.

[13] Rasoanirina B. N. V., Lassoued M. A., Kamoun A., Bahloul B., Miladi K., Sfar S. (2020). Voriconazole-loaded self-nanoemulsifying drug delivery system (SNEDDS) to improve transcorneal permeability. *Pharmaceutical Development and Technology*.

[14] Austin A., Lietman T., Rose-Nussbaumer J. (2017). Update on the management of infectious keratitis. *Ophthalmology*.

[15] Ghannoum M. A., Kuhn D. (2002). Voriconazole--better chances for patients with invasive mycoses. *European Journal of Medical Research*.

[16] Kalaiselvi G., Narayana S., Krishnan T., Sengupta S. (2015). Intrastromal voriconazole for deep recalcitrant fungal keratitis: a case series. *British Journal of Ophthalmology*.

[17] Prakash G., Sharma N., Goel M., Titiyal J. S., Vajpayee R. B. (2008). Evaluation of intrastromal injection of voriconazole as a therapeutic adjunctive for the management of deep recalcitrant fungal keratitis. *American Journal of Ophthalmology*.

[18] Nejabat M., Yaqubi N., Khosravi A., Zomorodian K., Ashraf M., Salouti R. (2016). Therapeutic effect of intrastromal voriconazole, topical voriconazole, and topical natamycin on Fusarium keratitis in rabbit. *Journal of ophthalmology*.

[19] Konar P., Joshi S., Mandhare S. J., Thakur R., Deshpande M., Dayal A. (2020). Intrastromal voriconazole: an adjuvant approach for recalcitrant mycotic keratitis. *Indian Journal of Ophthalmology*.

[20] Narayana S., Krishnan T., Ramakrishnan S. (2019). Mycotic antimicrobial localized injection. *Ophthalmology*.

[21] Solanki S., Rathi M., Khanduja S., Dhull C., Sachdeva S., Phogat J. (2015). Recent trends: medical management of infectious keratitis. *Oman Journal of Ophthalmology*.

[22] Cho C. H., Lee S.-B. (2018). Comparison of clinical characteristics and antibiotic susceptibility between *Pseudomonas aeruginosa* and P. *Putida keratitis* at a tertiary referral center: a retrospective study. *BMC Ophthalmology*.

[23] Wilhelmus K. R., Gee L., Hauck W. W. (1994). Herpetic eye disease study. *Ophthalmology*.

[24] Prajna N. V., Krishnan T., Mascarenhas J. (2013). The mycotic ulcer treatment trial. *JAMA Ophthalmology*.

[25] Schulze-Bonsel K., Feltgen N., Burau H., Hansen L., Bach M. (2006). Visual acuities “hand motion” and “counting fingers” can be quantified with the Freiburg visual acuity test. *Investigative Ophthalmology & Visual Science*.

[26] Srinivasan M., Mascarenhas J., Rajaraman R (2012). Corticosteroids for bacterial keratitis. *Archives of Ophthalmology*.

[27] Li-Xin X., Wei-Yun S., Xiao-Guang D. (1999). Clinical and histopathological study of fungal keratitis in 108 cases. *Chinese Ophthalmic Research*.

[28] Han S. B., Shin Y. J., Hyon J. Y., Wee W. R. (2011). Cytotoxicity of voriconazole on cultured human corneal endothelial cells. *Antimicrobial Agents and Chemotherapy*.

[29] Park C. H., Lee H. S., Chung S. K. (2014). Toxicity of intrastromal voriconazole injection on corneal endothelium in rabbits. *Cornea*.

[30] Hariprasad S. M., Mieler W. F., Holz E. R (2004). Determination of vitreous, aqueous, and plasma concentration of OrallyAdministered voriconazole in humans. *Archives of Ophthalmology*.

[31] Marangon F. B., Miller D., Giaconi J. A., Alfonso E. C. (2004). In vitro investigation of voriconazole susceptibility for keratitis and endophthalmitis fungal pathogens. *American Journal of Ophthalmology*.

[32] Niu L., Liu X., Ma Z (2020). Fungal keratitis: pathogenesis, diagnosis and prevention. *Microbial Pathogenesis*.

[33] Sav H., Ozdemir H. G., Altınbas R., Kiraz N., Ilkit M., Seyedmousavi S. (2016). Virulence attributes and antifungal susceptibility profile of opportunistic fungi isolated from ophthalmic infections. *Mycopathologia*.

[34] Ballal S., Belur S., Laha P., Roy S., Swamy B., Inamdar S. R. (2017). Mitogenic lectins from Cephalosporium curvulum (CSL) and Aspergillus oryzae (AOL) mediate host–pathogen interactions leading to mycotic keratitis. *Molecular and Cellular Biochemistry*.

[35] Niki M., Eguchi H., Hayashi Y., Miyamoto T., Hotta F., Mitamura Y. (2014). Ineffectiveness of intrastromal voriconazole for filamentous fungal keratitis. *Clinical Ophthalmology (Auckland, NZ)*.

[36] Tu E. Y. (2009). Alternaria keratitis: clinical presentation and resolution with topical fluconazole or intrastromal voriconazole and topical caspofungin. *Cornea*.

